# Senegenin regulates the mechanism of insomnia through the Keap1/Nrf2/PINK1/Parkin pathway mediated by GAD67

**DOI:** 10.1111/jsr.14354

**Published:** 2024-10-08

**Authors:** Honglin Jia, Xu Chen, Zhengting Liang, Ruining Liang, Jinhong Wu, Yanling Hu, Wenjun Cui, Xingping Zhang

**Affiliations:** ^1^ Xinjiang Medical University Fourth Clinical Medical College Urumqi China; ^2^ Xinjiang Medical University Urumqi China; ^3^ Affiliated Hospital of Traditional Chinese Medicine of Xinjiang Medical University Urumqi China

**Keywords:** GAD67, insomnia, Keap1/Nrf2, lentivirus, Parkin/PINK1

## Abstract

GAD67 impacts insomnia as a key enzyme catalysing the conversion of glutamate (Glu) to gamma‐aminobutyric acid (GABA). Senegenin enhances neuroprotection and is used widely to treat insomnia and other neurological diseases. This study aimed to investigate how senegenin regulates insomnia through a GAD67‐mediated signalling pathway. We measured GAD67 expression levels in insomnia patients and evaluated the expression levels of GAD67 and Keap1/Nrf2/Parkin/PINK1‐related cytokines following GAD67 lentiviral transfection in PC12 cells and in rat models. We also assessed cellular reactive oxygen species (ROS) and mitochondrial membrane potential levels. Additionally, EEG/EMG was used to analyse the sleep phases of rats and to assess memory and exploration functions. Pathological changes and the expression of GAD67 and sleep‐related proteins in the hippocampus were examined. The results showed that GAD67 expression was increased in insomnia patients, ROS levels were elevated, and the mitochondrial membrane potential was decreased in the GAD67‐KD group. Insomnia rats exhibited changes in sleep rhythm, learning, and exploration dysfunction, pathological changes in the CA1 region of the hippocampus, and differential expression of GAD67 and sleep‐related factors. Inhibitory neurofactor expression levels were decreased in insomnia rats, showing a positive correlation in the GAD67‐KD group and a negative correlation in the GAD67‐OE group. Conversely, excitatory factor expression levels were increased in insomnia rats, showing a positive correlation in the GAD67‐KD group and a negative correlation in the GAD67‐OE group. Senegenin intervention modulated cytokine expression levels. In conclusion, GAD67 negatively regulates insomnia, and senegenin can regulate insomnia by mediating the expression of cytokines in the GAD67‐regulated Keap1/Nrf2/Parkin/PINK1 pathway.

## 1 INTRODUCTION

Insomnia is a common neurological disease worldwide, mainly defined as continuous sleep disorders that are unsatisfactory for sleep quality and that affect daytime social function (Sutton, 2021; Van Someren, 2021), and the global incidence is increasing year by year, with about one‐third of the adult population and one‐half of the elderly population worldwide suffering from insomnia (Perlis et al., 2022; Sexton et al., 2020). Long‐term insomnia can seriously affect the quality of life and body function and is a risk factor for the development of mental disorders or other medical diseases (Buysse, 2013; Ebben & Kapella, 2014). Currently, effective treatments for insomnia include cognitive behavioural therapy (CBT‐I) and pharmacotherapy (Alimoradi et al., 2022): CBT‐I has advantages in safety and durability, but accessibility and cost‐effectiveness issues have limited it due to a shortage of medical human resources (Kay‐Stacey & Attarian, 2016; Riemann et al., 2023; Riemann & Perlis, 2009); benzodiazepines for the γ‐aminobutyric acid receptor are used widely for insomnia, but dependence and safety issues remain of concern (Cunnington et al., 2013). Treatment of insomnia with Chinese herbal medicine has fewer side effects, no withdrawal symptoms, and significant effects, and is increasingly used in clinical applications. Senegenin (SEN) is the main active compound extracted from the rhizome of *Polygala tenuifolia* (the plant name has been checked with http://www.worldfloraonline.org, ID: wfo‐0000488123), which has various pharmacological effects such as antioxidant, anti‐inflammatory, inhibition of neuronal apoptosis, and enhancement of neuroprotection and is widely used in the treatment of neurological diseases.

**FIGURE 1:**
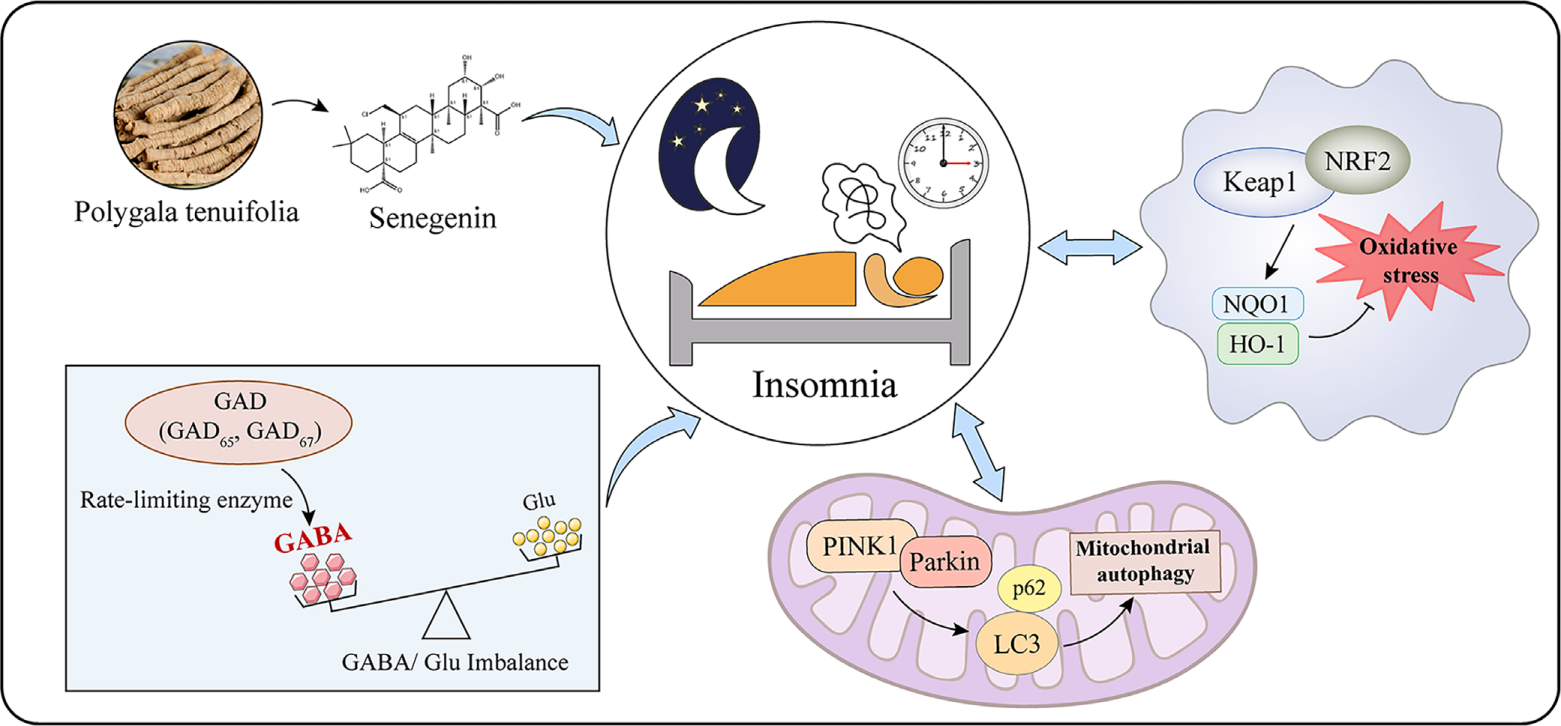
Senegenin mediates the Keap1/Nrf2/Parkin/PINK1 pathway to regulate insomnia through GAD67.

The pathogenesis of insomnia is complex and inhibitory/excitatory neurotransmitter imbalance plays a central role (Scammell et al., 2017). The GABAergic system is one of the effective mechanisms regulating insomnia (Avidan & Neubauer, 2017), and enhancing γ‐aminobutyric acid (GABA)‐mediated neuroinhibition shows a good potential to regulate insomnia. Glutamate decarboxylase 67 (GAD67) is the rate‐limiting enzyme that converts glutamate (Glu) into GABA and plays an important role in GABA production and GABAergic neuron development (Bolneo et al., 2022). It has been shown that activation of the GABAergic system facilitates sleep (Kim et al., 2019), and up‐regulation of GAD67 expression further promotes GABAergic system pathways, thereby increasing NREM sleep (Choi et al., 2014). In recent years, starting from epigenetics, exploring the mechanism of insomnia at the genetic level has become a hot topic. Due to the importance of GAD67 in its clinical, pharmacological, and genetic mutations, more and more attention has been paid to establishing genetic models to study the disease mechanism. Tamamaki's study found that the decreased expression of GAD67 in GAD^+/−^ mice resulted in impaired GABAergic development, persistent defects in synaptic transmission, and triggered inhibitory/excitatory transmitter imbalance (Tamamaki et al., 2003); however, Kuwana's study found that GABA synthesis was reduced by 95% in GAD^−/−^ mice, resulting in death at birth due to respiratory failure (Kuwana et al., 2003). Therefore, considering better safety, we chose GAD67 expression in viral vectors to explore how senegenin mediates the Keap1/Nrf2/Parkin/PINK1 pathway to regulate insomnia through GAD67 (Figure 1).

## 2 MATERIALS AND METHODS

### 2.1 Ethics statement

Our study was approved by the ethics committee at the Xinjiang Medical University (Batch number: IACUC‐2021011507). The informed consent was signed by all subjects. The animal experiments were approved by the Experimental Animal Ethics Committee of the Xinjiang Medical University (Certificate number: SYXK2023‐0002). All experimental procedures were performed under the International Code of Ethics, ARRIVE guidelines, and the National Research Council's Guide for the Care and Use of Laboratory Animals.

### 2.2 Patients

Forty‐four patients were between 20 and 80 years old and had presented with a diagnosis of insomnia. The patients were treated at the Traditional Chinese Medicine Hospital of the Xinjiang Uygur Autonomous Region from January 2022 to February 2023. These patients were clinically diagnosed based on the Diagnostic and Statistical Manual of Mental Disorders. Insomnia symptoms met the diagnostic criteria for at least 1 month and occurred at least three times a week at the time of diagnosis. At the same time, 44 volunteers of similar age without insomnia symptoms were also recruited as normal controls. Patients were excluded from the study in the following situations: presence of other sleep disorders, mental health disorders, substance abuse or dependence, shift work or irregular sleep patterns, and cognitive impairment.

### 2.3 PC12 cells transfected with GAD67 lentivirus

The rat adrenal pheochromocytoma cell line PC12 was obtained from the Cell Resource Centre of the Shanghai Institute of Biological Sciences, Chinese Academy of Sciences, and no contamination with other cell lines/mycoplasmas was identified by STR profiling. Cells were cultured in DMEM medium (Gibco) supplemented with 10% heat‐inactivated fetal bovine serum (FBS; Gibco) and 1% penicillin–streptomycin solution at 37°C in an atmosphere of 5% CO_2_. For transfection of these cells, GAD67‐knockdown lentivirus (GAD67‐KD; #SH673; GenePharma; sequence 5′ to 3′: TTGCAGACATATGTGAGAAAT), GAD67‐overexpressing lentivirus (GAD67‐OE; #SH5573; GenePharma; sequence 5′ to 3′: see supporting materials), and negative control lentivirus (Control; #SH678; GenePharma; sequence 5′ to 3′: TTCTCCGAACGTGTCACGT) at a titre of 1 × 10^8^ TU/mL, diluted in Opti‐MEM (#31985062; Invitrogen) according to the manufacturer's instructions and transfected.

#### 2.3.1 Mitochondrial membrane potential assay

PC12 cells were seeded in six‐well plates 1 day in advance at 5 × 10^5^ cells per well. When the cells grew to log phase, GAD67 lentiviral transfection was performed according to the experimental groups, and 72 h after transfection, the mitochondrial membrane potential was measured (#C20038; Beyotime). The culture medium was removed by aspiration, the cells were washed with PBS, 1 mL cell culture medium and 1 mL JC‐1 working solution were added, mixed thoroughly, and incubated in a cell incubator at 37°C for 20 min; the supernatant was removed by aspiration, washed twice with JC‐1 staining buffer, and 2 mL cell culture medium was added for observation under a confocal laser scanning microscope.

### 2.4 Insomnia rats transfected with GAD67 lentivirus

#### 2.4.1 Experimental animals and groups

Sixty specific pathogen‐free male Sprague–Dawley rats (aged 5–6 weeks, 180–200 g) were purchased from the Animal Experimental Centre of Xinjiang Medical University (Urumqi, China). All rats were housed in a clean barrier environment under standard conditions, including 12 h light/dark cycles, temperature (18–22°C), and humidity (50%–60%), with food and water ad libitum. The mice were randomly divided into six groups of 10 rats each and grouped as follows: (a) normal group; (b) insomnia group; (c) GAD67‐KD group; (d) KD + SEN group; (e) GAD67‐OE group; (f) OE + SEN group.

#### 2.4.2 Insomnia model establishment and GAD67 lentiviral transfections

The insomnia group was injected subcutaneously with 350 mg/kg/d DL‐4‐chlorophenylalanine (PCPA; #C136727; Aladdin) for 3 consecutive days to establish an insomnia model. The GAD67‐KD and GAD67‐OE groups were given in situ injections of GAD knockdown and overexpression lentiviruses with a titre of 1 × 10^9^ daily from days 4 to 10 based on the insomnia group. According to the requirements of the National Standards for the Enactment of traditional Chinese medicine (TCM, WS3‐B‐2769‐97) for the identification of TCM compounds containing *Polygala tenuifolia*, it is known that the usual dose of *Polygala tenuifolia* is 10 g/day for a 70 kg adult, which contains at least 26 mg senegenin. It can be seen that the daily intake of senegenin in rats should be 2.3 μg/g. Therefore, in this study, the KD + SEN and OE + SEN groups were administered high‐dose senegenin intervention 2 h after lentiviral intervention from days 4 to 10 to rats based on the lentiviral intervention group (9.2 μg/g; IS0610; Solarbio; HPLC ≥98%). Rats in the normal group were intragastrically administered with 0.9% sodium chloride at the same schedule. The rats were killed with 2% pentobarbital on the first day after the intervention in each group. The brain tissue was collected and stored at −80°C for future experiments.

#### 2.4.3 Criteria for modelling insomnia rats by EGG/EMG

Twenty rats were randomly divided into two groups: the normal group and the insomnia group. Rats in both groups received intraperitoneal injections of 30 mg/kg pentobarbital sodium and underwent surgery to instal a head mount with electrodes. Following the surgery, the rats were administered penicillin (2 × 10^5^ U/d) for 3 consecutive days. After a recovery period of 1 week, EEG/EMG recordings were conducted to assess the sleep phases of the rats. EEG/EMG analysis was performed in this study to determine the successful establishment of the rat insomnia model.

#### 2.4.4 Morris water maze test

After the intervention, all rats underwent a place navigation test. We placed a hidden transparent platform 2 cm below the water surface in the centre of a circular pool with a diameter of 200 cm and a height of 60 cm. During the experiment, the rats were placed facing the pool wall from the midpoint of each quadrant, with a 15 s interval between placements. The time taken by the rats to find the platform within 90 s and the swimming path were recorded as the escape latency and the total distance travelled, respectively. If a rat failed to locate the platform within 120 s, it was guided to stay on the platform for 10 s. The escape latency was then recorded as 90 s. This process was repeated for 4 consecutive days, and the average escape latency and the total distance travelled on day 4 were used to evaluate the learning and memory abilities of the rats.

#### 2.4.5 Open field test

The open field experiment utilised a chamber measuring ~30–40 cm in height and 100 cm in base length, with the surrounding walls coloured black. The floor of the open field was evenly divided into 25 square sections measuring 4 × 4 cm each. The laboratory environment was maintained in a quiet condition with a temperature of around 20°C and ample lighting. The rats were placed at the centre of the grid, and simultaneous recording and timing were conducted for 5 min. A computerised tracking analysis system was employed to assess the activity status of the rats over a specified period. Observed parameters included the number of crossings between grid squares, instances of rearing, time spent in the central square, and the number of crossings through the central square.

#### 2.4.6 Immunofluorescence

Frozen sections of rat hippocampal tissue were heated at 60°C for 2 h and dehydrated with graded ethanol. Antigen retrieval was performed with 1 × Tris‐EDTA (#C1038; Solarbio) at 100°C for 5 min. Membranes were then permeabilised with 0.1% Triton X‐100 (#T8200; Solarbio) for 30 min and incubated with primary antibodies (GAD67 – #sc‐28376, 1:100, Santa Cruz) overnight at 4°C in a humidified chamber. The following day, slides were washed three times with 1 × PBST and incubated with secondary antibodies (#SA00003‐2; Proteintech) for 20 min at 25°C. The sections were then washed three times with 1 × PBST and sealed with an anti‐fluorescent quenching mounting medium containing DAPI (#G1407; Servicebio), and images were collected for analysis at a confocal laser scanning microscope.

#### 2.4.7 Haematoxylin and eosin (HE) staining

The rat hippocampal tissue was subjected to paraffin embedding and sectioning at 60°C for 2 h. The sections underwent gradient alcohol dehydration followed by staining with haematoxylin and eosin (HE) for 5 min. After differentiation with hydrochloric acid, the sections were stained with eosin and counterstained with ammonia water. Subsequently, gradient alcohol was used for the dehydration of the slides, which were then sealed with neutral gum. Pathological changes in the hippocampal tissue were observed under a light microscope, and images were captured.

#### 2.4.8 Immunohistochemistry (IHC) staining

Paraffin sections of rat hippocampal tissue were heated at 60°C for 2 h and dehydrated with graded ethanol. Antigen retrieval was performed with 1 × Tris‐EDTA (#C1038; Solarbio) for 5 min at 100°C. The tissues were then blocked with 1% bovine serum albumin (BSA; #A8010; Solarbio) for 30 min at 25°C and incubated with primary antibodies (GAD_65/67_ – #sc‐365,180, 1:500, Santa Cruz; GABA‐T – #11349‐1‐AP, 1:100, Proteintech; BDNF – #28205‐1‐AP, 1:500, Proteintech; GluR_2_ – #bs‐1798R, 1:300, Bioss; 5‐HT_1A_R – #bs‐1124R, 1:300, Bioss; 5‐HT_2A_R – #bs‐1056R, 1:300, Bioss) in a humid chamber overnight at 4°C. The next day, the slides were washed three times with 1 × PBST and incubated with secondary antibodies (Goat Anti‐Rabbit IgG polymer – #PV‐9001; ZSGB‐BIO) for 20 min at 25°C. They were then washed three times with 1 × PBST and incubated with a DAB chromogenic dilution (#ZLI‐9018; ZSGB‐BIO) for 1 min. The sections were sealed with neutral gum after haematoxylin staining. Protein distribution in the hippocampal tissue was observed under a light microscope, and images were collected for analysis.

### 2.5 Enzyme‐linked immunosorbent assay (ELISA)

Blood serum from the patients was centrifuged at 3000 rpm for 30 min and stored at −80°C. The levels of cytokine GAD67 in patients were measured using ELISA kits (#YX‐070105H; Sino Best Biological) according to the manufacturer's instructions. The absorbance wavelength of A450_nm_ was measured using a microplate reader, a standard curve was constructed, and the concentration value of each sample was calculated.

### 2.6 Reverse transcription quantitative real‐time polymerase chain reaction (RT‐qPCR)

Total RNA from rat brain tissue and PC12 cells was extracted using TRIzol reagent (#15596018; Thermo Fisher Scientific) according to the manufacturer's instructions. The RNA concentration was measured, and the reverse transcription reaction was performed using PrimeScript RT reagent kit with gDNA Eraser (#RR047A; Takara) according to the 500 ng total RNA content of each group. The RT‐qPCR reaction solution was prepared according to TB Green Premix Ex Taq II (#RR820A; Takara) instructions. The reaction solution was centrifuged and mixed in a MyCyclerTM Thermal Cycler for amplification reaction and analysis. GAPDH was used as an internal reference, and the experiment was repeated three times. The relative expression of mRNA was calculated according to the 2^−ΔΔCT^ method. The primer sequences used in the experiments are listed in Table 1.

**TABLE 1:** Detailed information about primer sequence.

Gene	Sequence (5′ to 3′) – forward	Sequence (5′ to 3′) – reverse
GAD67	CTTCCACCACCCACACCAGT	TTGTACCGAGCCGCCATGAT
GABA‐T	GACCTGCTCAACAACGTGGC	ATCCCTGAAGACCAGCGTGG
BDNF	TGTGGTCAGTGGCTGGCTCTC	ACAGGACGGAAACAGAACGAACAG
GluR_2_	ACACTGCAAGCTGTTCTGGA	TGTGTGTGCTCCAGGGTATT
5‐HT_1A_	AAGTTCTGCCGCCGATGATGATG	TCCTCCTCTTCCTCCTCCTCCTC
5‐HT_2A_	CCGCTATGTCGCCATCCAGAAC	ACAGATATGGTCCACACGGCAATG
Keap1	TGCTCAACCGCTTGCTGTATGC	TCATCCGCCACTCATTCCTCTCC
Nrf2	GCCTTCCTCTGCTGCCATTAGTC	TGCCTTCAGTGTGCTTCTGGTTG
NQO1	AGGCTGCTGTGGAGGCTCTG	GCTCCCCTGTGATGTCGTTTCTG
HO‐1	CAGACAGAGTTTCTTCGCCAGAGG	TGTGAGGACCCATCGCAGGAG
PINK1	CCTCCAGCGAAGCCATCTTAAGC	GACTGTCTACCGCCTGAACTGTTG
Parkin	AATGGCCTGGGCTGTGGGTTTG	CCTGCTCGGCGGCTCTTTCATC
LC3	CCAGGACAAGCAGGCAGATGAAG	CAGGCTTTCGTCTCTTCCACCATC
p62	TCGTGGTCGTGGGGTGTCTG	TCTGGTGATGGAGCCTCTTACTGG
GAPDH	GACCTGACCTGCCGCCTA	AGGAGTGGGTGTCGCGCTGT

### 2.7 Western blotting (WB)

Total protein was extracted from the brain tissues of rats and PC12 cells using RIPA lysis buffer (#R0010; Solarbio) according to the manufacturer's instructions. Protein concentrations were quantified using the BCA protein assay kit (#PC0020; Solarbio). Equal amounts of protein were separated by 10% sodium dodecyl sulphate‐polyacrylamide gel electrophoresis and transferred onto polyvinylidene fluoride membranes. The membranes were then sealed with 5% skim milk for 2 h at 25°C and incubated with primary antibodies (GAPDH – #ab8245, 1:10000, Abcam; GAD67 – #sc‐28376, 1:300, Santa Cruz; GAD65 – #sc‐377145, 1:500, Santa Cruz; GAD_65/67_ – #sc‐365180, 1:500, Santa Cruz; GABA‐T – #11349‐1‐AP, 1:1500, Proteintech; Keap1 – #WL03285, 1:1500, Wanleibio; Nrf2 – #WL02135, 1:800, Wanleibio; NQO1 – #WL04860, 1:800, Wanleibio; HO‐1 – #WL02400, 1:1500, Wanleibio; PINK1 – #WL04963, 1:800, Wanleibio; Parkin – #WL02512, 1:500, Wanleibio; LC3 – #WL01506, 1:1500, Wanleibio; p62 – #WL02385, 1:1000, Wanleibio; BDNF – #28205‐1‐AP, 1:1000, Proteintech; GluR_2_ – #bs‐1798R, 1:1000, Bioss; 5‐HT_1A_R – #bs‐1124R, 1:1000, Bioss; 5‐HT_2A_R – #bs‐1056R, 1:1000, Bioss) overnight at 4°C. The next day, the membranes were washed three times with 1TBST and incubated with secondary antibodies (HRP‐IgG – #ZB‐2301, 1:40000, ZSGB‐BIO) for 1 h at 25°C. After washing three times with 1TBST, the membranes were exposed to enhanced chemiluminescence (ECL; #BL520A; Biosharp) chromogenic substrates, and the results were captured using a gel imaging system. The experiments were repeated in triplicate, and the grey values were analysed using ImageJ software.

### 2.8 Statistical analysis

Statistical analysis was performed using SPSS software version 25.0 (IBM Corp.) and GraphPad Prism 9.0 (GraphPad Software Inc.). All data are presented as the mean ± standard deviation (SD). Differences between two groups were compared using an unpaired *t*‐test, and differences among multiple groups were compared using a one‐way analysis of variance (ANOVA). The value of *p* < 0.05 was considered statistically significant.

## 3 RESULTS

### 3.1 Expression pattern of GAD67 in insomnia patients

**FIGURE 2:**
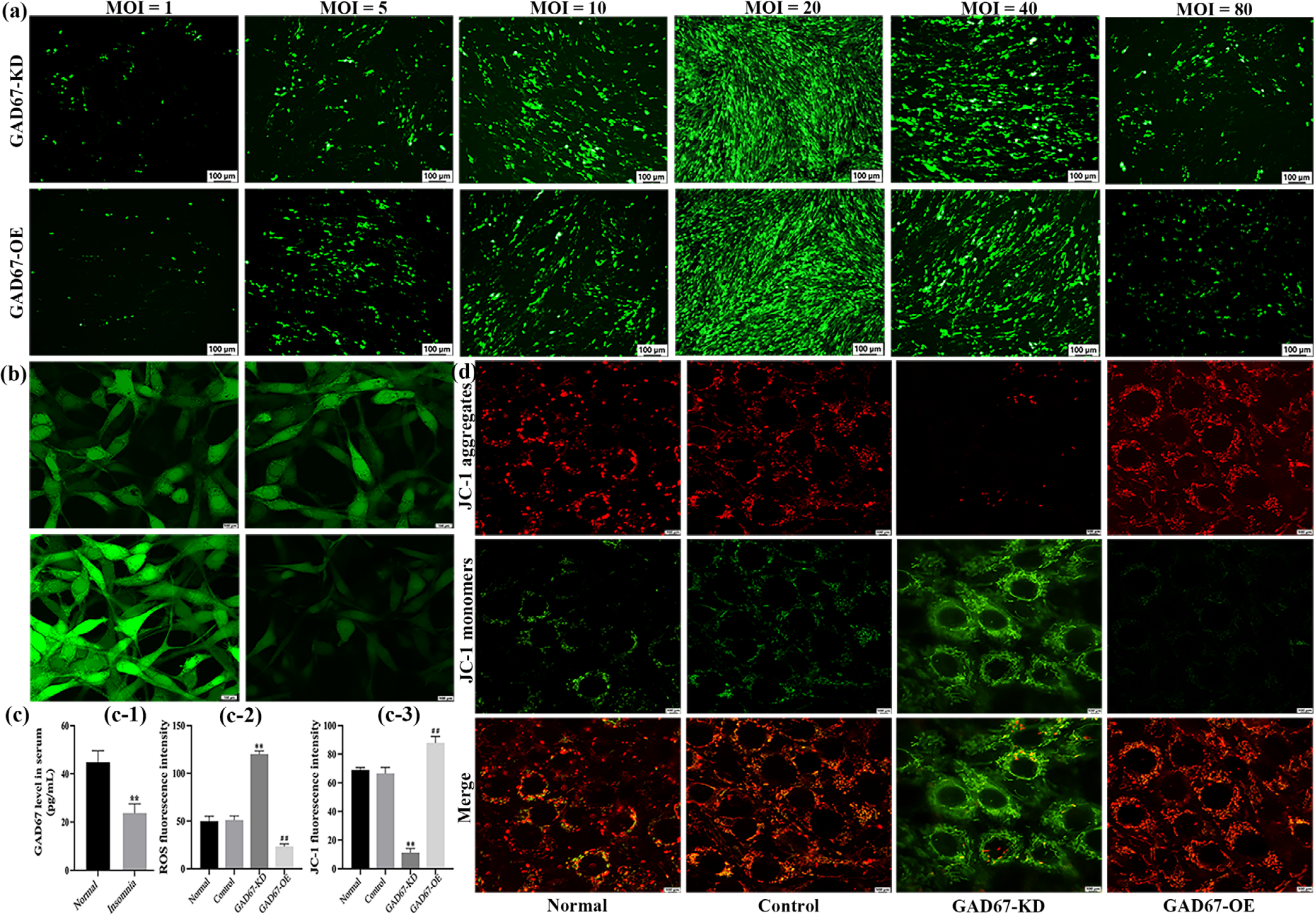
(a) Infestation efficiency of PC12 cells transfected with GAD67 lentiviruses (100×). (b) Expression levels of ROS in PC12 cells transfected with GAD67 lentiviruses (200×). (c‐1) Histogram of GAD67 expression in insomnia patients. (c‐2) Histogram of ROS expression levels in GAD67 lentivirus‐transfected cells. (c‐3) Histogram of JC‐1 fluorescence intensity in GAD67 lentivirus‐transfected cells. (d) Expression of JC‐1‐labelled mitochondrial membrane potential in GAD67 lentivirus transfected cells (400×). The results are expressed as mean ± SD, and the experiments were repeated three times; **p < 0.01 versus normal group; ^##^ *p* < 0.01 versus GAD67‐KD group; the difference was statistically significant.

The expression of GAD67 in the serum of normal and insomnia patients was determined by ELISA. The results showed that the expression of GAD67 in the insomnia group was significantly lower than that in the normal group (*p* < 0.01) (Figure 2c‐1).

### 3.2 Transfection expression of GAD67 lentivirus in PC12 cells

The infection efficiency of GAD67 lentivirus transfected PC12 cells was determined by the multiplicity of infection (MOI), and the lentiviral titre with the MOI value with the best infection efficiency was selected as the transfection concentration. As shown in Figure 2a, the optimal MOI values in PC12 cells transfected with GAD67‐KD and GAD67‐OE lentiviruses were 20. Therefore, we chose a viral titre of MOI = 20 as the transfection concentration for subsequent experiments.

### 3.3 ROS content assay in PC12 cells

Confocal laser scanning was used to detect ROS expression levels in PC12 cells transfected with GAD67. As shown in Figure 2b, the ROS expression levels in the GAD67‐KD group were significantly increased compared with the normal group (*p* < 0.01), and ROS expression levels in the GAD67‐OE group were significantly decreased compared with the GAD67‐KD group (*p* < 0.01). In addition, there was no statistical difference in the expression level of ROS between the control group and the normal group (*p* > 0.05) (Figure 2c‐2).

### 3.4 Mitochondrial membrane potential expression in PC12 cells

**FIGURE 3:**
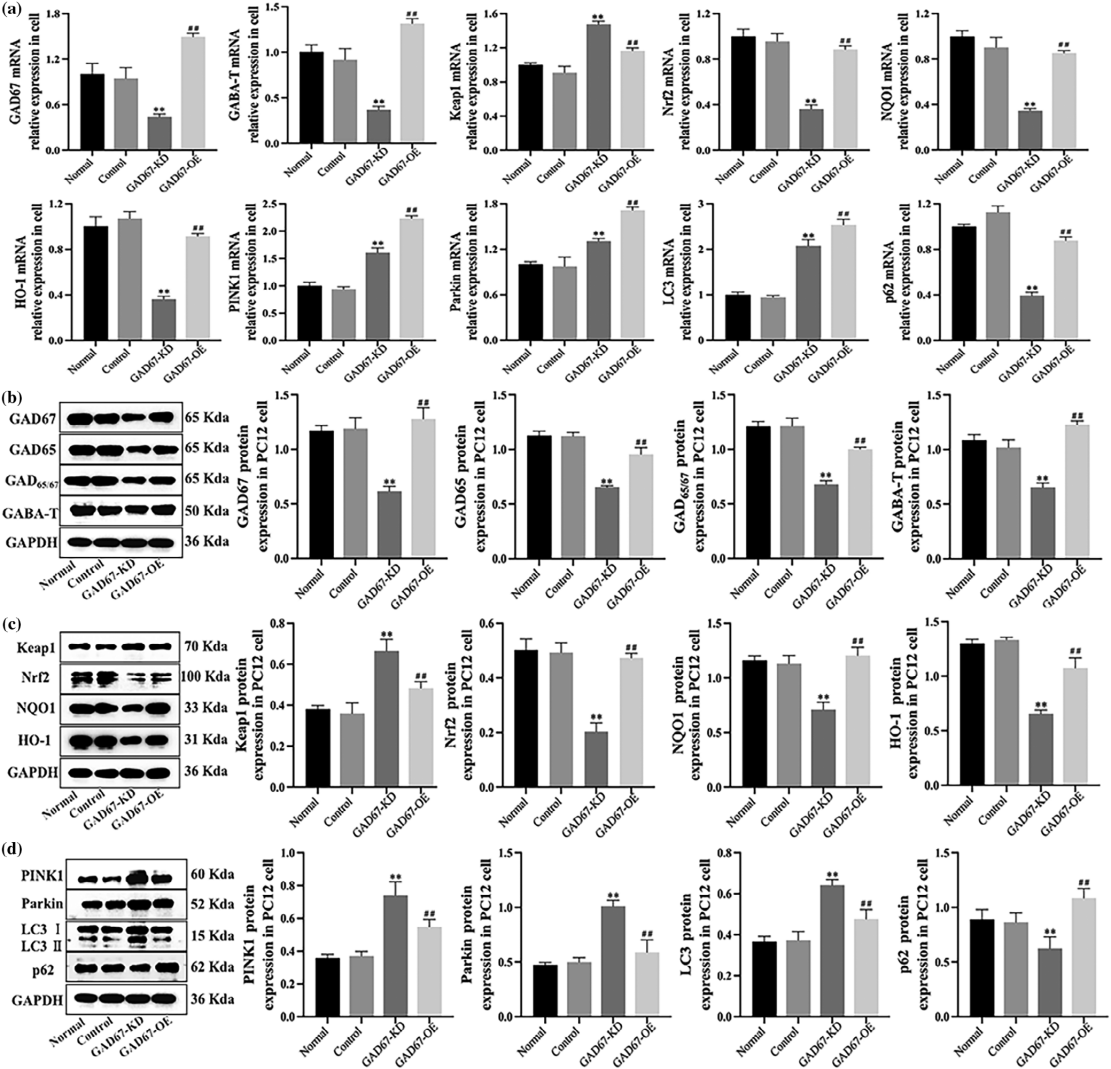
(a) mRNA expression patterns of GAD67 and pathway cytokines in PC12 cells. (b) Strip image and protein expression levels of GAD67, GAD65, GAD_65/67_, and GABA‐T in PC12 cells. (c) Strip image and protein expression levels of Keap1, Nrf2, NQO1, and HO‐1 in PC12 cells. (d) Strip image and protein expression levels of PINK1, Parkin, LC3, and p62 in PC12 cells. GAPDH was used as an internal reference, and the experiments were repeated three times. Results are expressed as mean ± SD; **p < 0.01 versus normal group; ^##^ *p* < 0.01 versus GAD67‐KD group; the difference is statistically significant.

A JC‐1 fluorescent probe was used to label mitochondria to reflect the damage of mitochondria, and the fluorescence intensity of cells was positively correlated with the expression level of mitochondrial membrane potential. As shown in Figure 2d, the fluorescence intensity of JC‐1 in the GAD67‐KD group was significantly lower than that in the normal group (*p* < 0.01), and the fluorescence intensity in the GAD67‐OE group was significantly higher than that in the GAD67‐KD group (*p* < 0.01). There was no statistically significant difference in JC‐1 expression between the normal and control groups (*p* > 0.05) (Figure 2c‐3).

### 3.5 mRNA expression patterns of GAD67 and pathway cytokines in PC12 cells

RT‐qPCR was performed to detect the mRNA expression of GAD67 and pathway cytokines in PC12 cells transfected with GAD67 lentivirus. As shown in Table 2, the mRNA expression levels of GAD67, GABA‐T, Nrf2, NQO1, HO‐1, and p62 were significantly decreased in the GAD67‐KD group compared with the normal group (*p* < 0.01), and the expression was significantly increased after GAD67‐OE intervention (*p* < 0.01). Compared with the normal group, Keap1 mRNA was significantly increased in the GAD67‐KD group (*p* < 0.01), and expression was significantly decreased after the GAD67‐OE intervention (*p* < 0.01). The mRNA expression levels of PINK1, Parkin, and LC3 were significantly increased in the GAD67‐KD group compared with the normal group (*p* < 0.01), and the expression was increased after GAD67‐OE intervention (*p* < 0.01). In addition, mRNA expression did not differ between the normal and control groups (Figure 3a).

**TABLE 2:** mRNA expression patterns of GAD67 and pathway cytokines in PC12 cells.

Group	Normal	Control	GAD67‐KD	GAD67‐OE
GAD67	1.01 ± 0.14	0.95 ± 0.14	0.44 ± 0.04**	1.50 ± 0.04^##^
GABA‐T	1.00 ± 0.08	0.92 ± 0.12	0.37 ± 0.04**	1.32 ± 0.05^##^
Keap1	1.00 ± 0.02	0.91 ± 0.07	1.48 ± 0.03**	1.16 ± 0.03^##^
Nrf2	1.00 ± 0.06	0.96 ± 0.07	0.36 ± 0.03**	0.89 ± 0.03^##^
NQO1	1.00 ± 0.05	0.90 ± 0.09	0.35 ± 0.02**	0.85 ± 0.02^##^
HO‐1	1.00 ± 0.09	1.07 ± 0.11	0.36 ± 0.03**	0.92 ± 0.02^##^
PINK1	1.00 ± 0.06	0.94 ± 0.04	1.61 ± 0.09**	2.24 ± 0.05^##^
Parkin	1.00 ± 0.04	0.97 ± 0.12	1.31 ± 0.03**	1.72 ± 0.04^##^
LC3	1.00 ± 0.06	0.94 ± 0.04	2.07 ± 0.14**	2.54 ± 0.12^##^
p62	1.00 ± 0.02	1.13 ± 0.05	0.40 ± 0.03**	0.88 ± 0.03^##^

*Note*: **p < 0.01 versus normal group; ^##^ *p* < 0.01 versus GAD67‐KD group; the difference is statistically significant.

### 3.6 Protein expression patterns of GAD67 and pathway cytokines in PC12 cells

We detected the protein expression of GAD67 and pathway cytokines after GAD67 lentiviral transfection of PC12 cells using Western blotting. As shown in Table 3, the protein expression levels of GAD67, GAD65, GAD_65/67_, GABA‐T, Nrf2, NQO1, HO‐1, and p62 in the GAD67‐KD group were significantly lower than those in the normal group (*p* < 0.01), and the expression was significantly increased after GAD67‐OE intervention (*p* < 0.01). Compared with the normal group, the protein levels of Keap1, PINK1, Parkin, and LC3 were significantly increased in the GAD67‐KD group (*p* < 0.01), and the expression was significantly decreased after the GAD67‐OE intervention (*p* < 0.01). There was no difference in protein expression between the normal and control groups (Figure 3b–d).

**TABLE 3:** Protein expression patterns of GAD67 and pathway cytokines in PC12 cells.

Group	Normal	Control	GAD67‐KD	GAD67‐OE
GAD67	1.17 ± 0.05	1.19 ± 0.10	0.62 ± 0.04**	1.28 ± 0.10^##^
GAD65	1.13 ± 0.04	1.12 ± 0.03	0.66 ± 0.01**	0.96 ± 0.06^##^
GAD_65/67_	1.21 ± 0.04	1.21 ± 0.07	0.68 ± 0.03**	1.00 ± 0.02^##^
GABA‐T	1.09 ± 0.05	1.02 ± 0.07	0.65 ± 0.04**	1.23 ± 0.03^##^
Keap1	0.38 ± 0.02	0.36 ± 0.06	0.66 ± 0.06**	0.48 ± 0.03^##^
Nrf2	0.50 ± 0.04	0.49 ± 0.04	0.20 ± 0.05**	0.47 ± 0.02^##^
NQO1	1.16 ± 0.04	1.13 ± 0.08	0.71 ± 0.07**	1.21 ± 0.08^##^
HO‐1	1.30 ± 0.04	1.33 ± 0.02	0.66 ± 0.03**	1.08 ± 0.09^##^
PINK1	0.36 ± 0.02	0.37 ± 0.03	0.74 ± 0.08**	0.55 ± 0.05^##^
Parkin	0.47 ± 0.02	0.50 ± 0.04	1.01 ± 0.05**	0.59 ± 0.11^##^
LC3	0.37 ± 0.03	0.37 ± 0.04	0.64 ± 0.03**	0.48 ± 0.05^##^
p62	0.89 ± 0.09	0.86 ± 0.09	0.62 ± 0.11**	1.09 ± 0.09^##^

*Note*: **p < 0.01 versus normal group; ^##^ *p* < 0.01 versus GAD67‐KD group; the difference is statistically significant.

### 3.7 EGG/EMG analysis in insomnia rats

**FIGURE 4:**
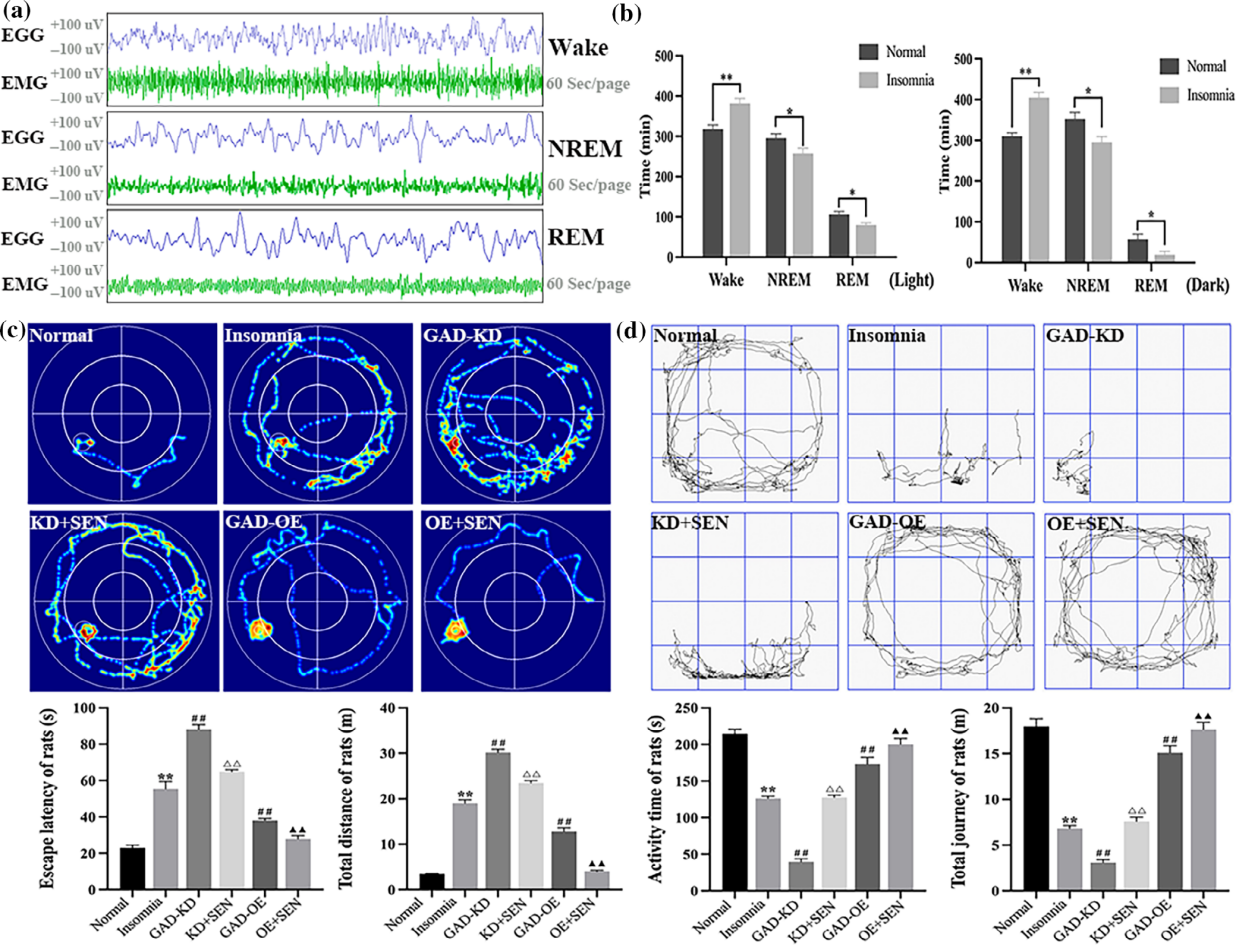
(a) Sleep band diagram of rats at each stage using EGG/EMG monitoring. (b) Histogram of sleep rhythm during light and dark in the normal group and insomnia group. (c) The motion tracking of Morris water maze in rats and the histogram of escape latency and total distance. (d) The exploration track of open field tests in rats and the histogram of activity time and total journey. Results are expressed as mean ± SD; *p < 0.05, **p < 0.01; **p < 0.01 versus normal group; ^##^ *p* < 0.01 versus Insomnia group; ^△△^ *p* < 0.01 versus GAD67‐KD group; ^▲▲^ *p* < 0.01 versus GAD67‐OE group; the difference is statistically significant.

EGG/EMG monitoring was used to analyse sleep phases in normal and insomnia rat groups. During the light and dark phases, the wake period was significantly prolonged (*p* < 0.01), the NREM period (*P* < 0.05), and the REM period (*p* < 0.05) were significantly reduced in the insomnia group compared with the control group. Therefore, this study successfully constructed a rat model of insomnia for subsequent experiments, as shown in Figure 4a,b.

### 3.8 Behavioural experiments in rats

In this study, rats were subjected to relevant behavioural experiments (Table 4). The Morris water maze showed that compared with the normal group, the escape latency and total distance of insomnia rats were significantly prolonged (*p* < 0.01), and the rats in the GAD67‐KD group had more impairment of learning cognitive ability, resulting in longer escape latency and total distance (*p* < 0.01), which were significantly shortened after senegenin intervention (*p* < 0.01). Compared with the insomnia group, the escape latency and total distance of rats in the GAD67‐OE group were significantly reduced (*p* < 0.01), the learning ability was enhanced, and the escape latency and total distance were shortened (*p* < 0.01) after senegenin intervention. The results of the open field test showed that the activity time and total journey of insomnia rats were significantly reduced compared with the normal group (*p* < 0.01), and the spatial exploration ability of GAD67‐KD rats was impaired, resulting in a further reduction of activity time and total journey (*p* < 0.01), which was significantly prolonged after senegenin intervention (*p* < 0.01). The activity time and the total journey of rats in the GAD67‐OE group were significantly longer than those in the insomnia group (*p* < 0.01). After senegenin intervention, the spatial exploration ability was enhanced, and the activity time and total journey were significantly increased (*p* < 0.01) (Figure 4c,d).

**TABLE 4:** Morris water maze and open field tests in rats.

Group	Normal	Insomnia	GAD67‐KD	KD + SEN	GAD67‐OE	OE + SEN
Escape latency (s)	23.03 ± 1.40	55.25 ± 4.23^**^	88.23 ± 2.45^**##**^	64.88 ± 1.07^△△^	38.13 ± 0.99^**##**^	27.80 ± 2.02^▲▲^
Total distance (m)	3.46 ± 0.13	18.99 ± 0.79^**^	30.16 ± 0.77^**##**^	23.44 ± 0.55^△△^	12.79 ± 0.84^**##**^	4.06 ± 0.20^▲▲^
Activity time (s)	214.60 ± 5.79	126.26 ± 2.91^**^	39.62 ± 4.11^**##**^	127.53 ± 3.01^△△^	173.53 ± 8.65^**##**^	200.12 ± 7.59^▲▲^
Total journey (m)	18.02 ± 0.77	6.75 ± 0.32^**^	2.96 ± 0.43^**##**^	7.62 ± 0.39^△△^	14.75 ± 1.07^**##**^	17.46 ± 0.84^▲▲^

*Note*: **p < 0.01 versus normal group; ^##^ *p* < 0.01 versus insomnia group; ^△△^ *p* < 0.01 versus GAD67‐KD group; ^▲▲^ *p* < 0.01 versus GAD67‐OE group; the difference is statistically significant.

### 3.9 HE pathology staining of brain tissue in rats

**FIGURE 5:**
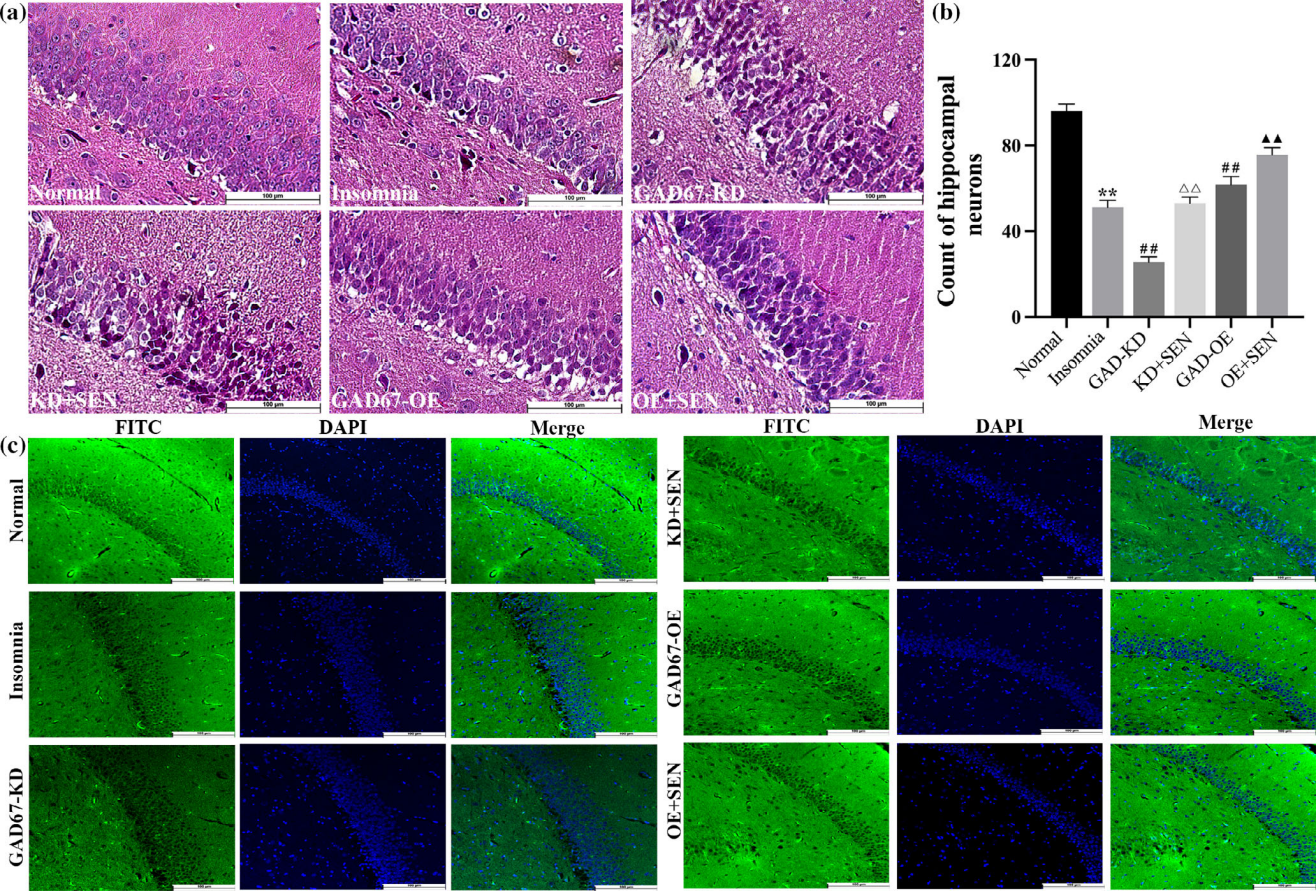
(a) HE staining of the hippocampus in the CA1 region of rats (200×). (b) Histogram of HE staining in hippocampal tissue. (c) The expression of GAD67 in the CA1 region of hippocampal tissue in rats was detected by IF (200×). Results are expressed as mean ± SD; **p < 0.01 versus normal group; ^##^ *p* < 0.01 versus insomnia group; ^△△^ *p* < 0.01 versus GAD67‐KD group; ^▲▲^ *p* < 0.01 versus GAD67‐OE group; the difference is statistically significant.

The morphology and integrity of neurons in the CA1 region of the hippocampus in rats were observed by HE pathological staining. As shown in Figure 5a,b, the hippocampal neurons in the CA1 region in the normal group were orderly arranged, with clear nuclei and intact structure, and the nuclei of the insomnia group were unclear, the intercellular spaces were enlarged, the neurons were arranged loosely, and the number of neurons was significantly reduced compared with the normal group (*p* < 0.01). In the GAD67‐KD group, the morphology of neurons in the CA1 region was changed, the cell membrane was ruptured, the nucleoplasm was difficult to distinguish, and the number of neurons was significantly decreased compared with the insomnia group (*p* < 0.01). After senegenin intervention, the morphology of neurons was slightly improved and the number was significantly increased in the KD + SEN group (*p* < 0.01), but there was still loose deformation. In the GAD67‐OE group, there was infiltration in the hippocampal tissue in the CA1 region, with cavities, looseness, and increased spaces, and after senegenin intervention, the degree of looseness was alleviated, the intercellular space was reduced, and the number of neurons was significantly increased (*p* < 0.01).

### 3.10 The expression of GAD67 in rat brain tissue was detected by immunofluorescence (IF)

**FIGURE 6:**
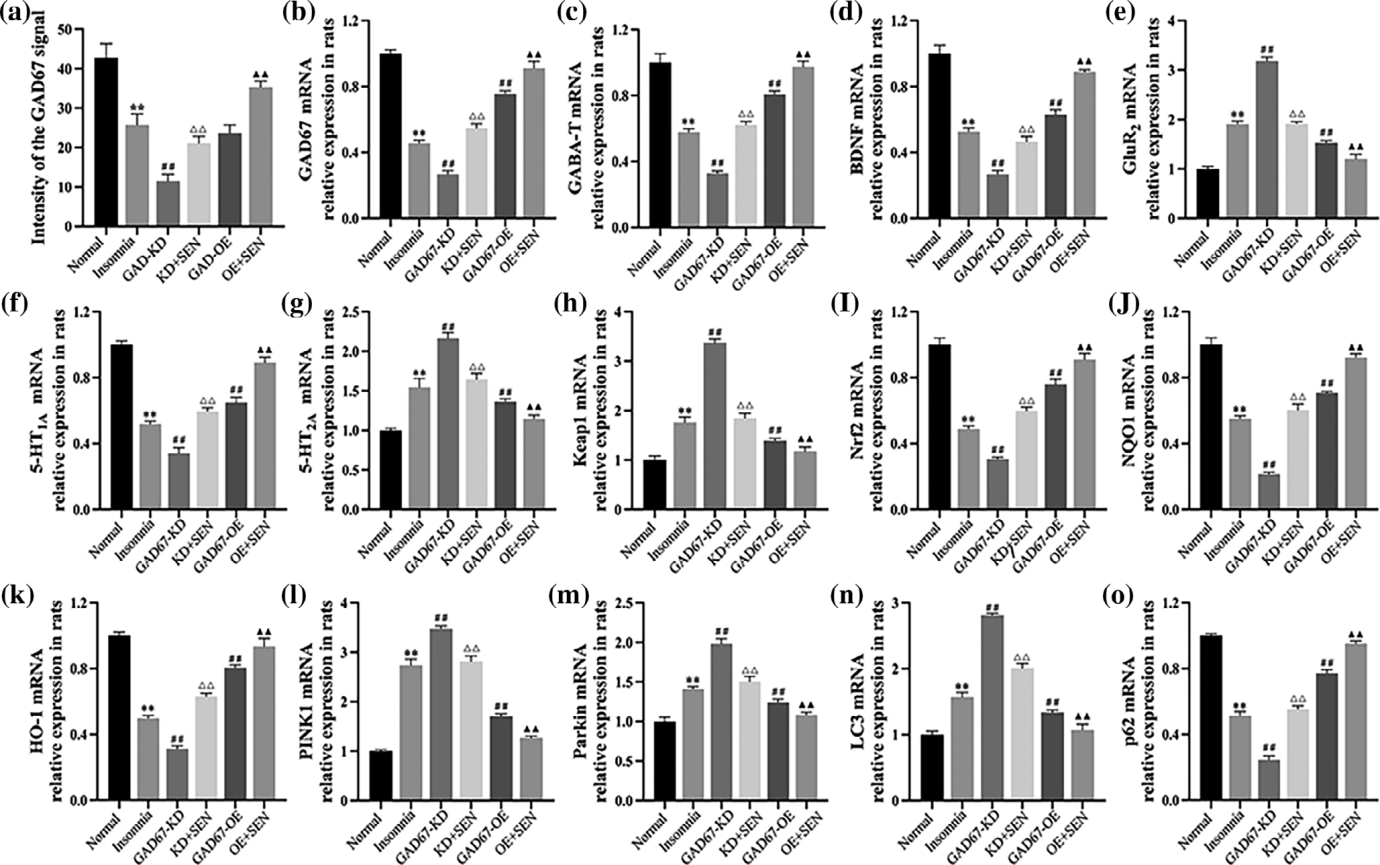
(a) Histogram of the expression of GAD67 in the CA1 region of hippocampal tissue in rats. The mRNA expression of (b) GAD67, (c) GABA‐T, (d) BDNF, (e) GluR_2_, (f) 5‐HT_1A_, (g) 5‐HT_2A_, (h) Keap1, (i) Nrf2, (j) NQO1, (k) HO‐1, (l) PINK1, (m) Parkin, (n) LC3, and (o) p62 in rats. GAPDH was used as an internal reference, and the experiments were repeated three times. Results are expressed as mean ± SD; **p < 0.01 versus normal group; ^##^ *p* < 0.01 versus insomnia group; ^△△^ *p* < 0.01 versus GAD67‐KD group; ^▲▲^ *p* < 0.01 versus GAD67‐OE group; the difference is statistically significant.

To further verify the protein level of GAD67 in the CA1 region of rat hippocampal tissue transfected with GAD67 lentivirus, IF was used to detect the expression and localisation of GAD67 in each group of rats. As shown in Figure 6a, in the CA1 region of the hippocampus, GAD67 expression was significantly decreased in the insomnia group compared with the normal group (*p* < 0.01). GAD67 expression was significantly decreased after GAD67‐KD transfection compared with the insomnia group (*p* < 0.01), and significantly increased after KD + SEN intervention (*p* < 0.01); there was no difference in expression between GAD67‐OE transfection and insomnia group, and the expression was significantly increased after OE + SEN intervention (*p* < 0.01) (Figure 5c).

### 3.11 mRNA expression patterns of GAD67 and pathway cytokines in rats

The expression levels of GAD67 and pathway cytokines were detected by RT‐qPCR after transfection with GAD67 lentiviral in rats. As shown in Table 5, mRNA expression levels of GAD67, GABA‐T, BDNF, 5‐HT_1A_, Nrf2, NQO1, HO‐1, and p62 were significantly decreased in insomnia rats (*p* < 0.01), further decreased after GAD67‐KD transfection (*p* < 0.01), and significantly increased after senegenin intervention (*p* < 0.01); the expression of GAD67‐OE was significantly increased after transfection (*p* < 0.01) and further increased after senegenin intervention (*p* < 0.01). Compared with the normal group, the mRNA expressions of GluR2, 5‐HT_2A_, Keap1, PINK1, Parkin, and LC3 in insomnia rats were significantly increased (*p* < 0.01), further increased after GAD67‐KD transfection (*p* < 0.01), and decreased after senegenin intervention (*p* < 0.01); the expression of GAD67‐OE was significantly decreased after GAD67‐OE transfection (*p* < 0.01) and further decreased after senegenin intervention (*p* < 0.01) (Figure 6).

**TABLE 5:** mRNA expression patterns of GAD67 and pathway cytokines in rats.

Group	Normal	Insomnia	GAD67‐KD	KD + SEN	GAD67‐OE	OE + SEN
GAD67	1.00 ± 0.02	0.46 ± 0.02^**^	0.27 ± 0.02^**##**^	0.55 ± 0.03^△△^	0.76 ± 0.02^**##**^	0.91 ± 0.04^▲▲^
GABA‐T	1.00 ± 0.05	0.58 ± 0.02^**^	0.33 ± 0.01^**##**^	0.62 ± 0.02^△△^	0.81 ± 0.02^**##**^	0.98 ± 0.06^▲▲^
BDNF	1.00 ± 0.05	0.53 ± 1.02^**^	0.27 ± 0.02^**##**^	0.46 ± 0.03^△△^	0.63 ± 0.03^**##**^	0.89 ± 0.01^▲▲^
GluR_2_	1.00 ± 0.05	1.90 ± 0.06^**^	3.18 ± 0.08^**##**^	1.92 ± 0.04^△△^	1.53 ± 0.04^**##**^	1.20 ± 0.09^▲▲^
5‐HT_1A_	1.00 ± 0.02	0.52 ± 0.02^**^	0.34 ± 0.03^**##**^	0.60 ± 0.02^△△^	0.65 ± 0.03^**##**^	0.89 ± 0.03^▲▲^
5‐HT_2A_	1.00 ± 0.01	1.55 ± 0.04^**^	2.16 ± 0.07^**##**^	1.64 ± 0.03^△△^	1.36 ± 0.01^**##**^	1.14 ± 0.05^▲▲^
Keap1	1.00 ± 0.08	1.76 ± 0.04^**^	3.38 ± 0.07^**##**^	1.85 ± 0.04^△△^	1.39 ± 0.02^**##**^	1.18 ± 0.03^▲▲^
Nrf2	1.00 ± 0.04	0.49 ± 0.02^**^	0.31 ± 0.01^**##**^	0.60 ± 0.02^△△^	0.76 ± 0.03^**##**^	0.91 ± 0.04^▲▲^
NQO1	1.00 ± 0.07	0.55 ± 0.02^**^	0.21 ± 0.01^**##**^	0.60 ± 0.04^△△^	0.71 ± 0.01^**##**^	0.92 ± 0.02^▲▲^
HO‐1	1.00 ± 0.02	0.50 ± 0.02^**^	0.31 ± 0.02^**##**^	0.63 ± 0.02^△△^	0.81 ± 0.02^**##**^	0.94 ± 0.02^▲▲^
PINK1	1.00 ± 0.03	2.74 ± 0.12^**^	3.47 ± 0.06^**##**^	2.82 ± 0.11^△△^	1.70 ± 0.05^**##**^	1.27 ± 0.04^▲▲^
Parkin	1.00 ± 0.05	1.41 ± 0.03^**^	1.98 ± 0.03^**##**^	1.51 ± 0.02^△△^	1.24 ± 0.04^**##**^	1.08 ± 0.03^▲▲^
LC3	1.00 ± 0.05	1.57 ± 0.07^**^	2.81 ± 0.03^**##**^	2.01 ± 0.07^△△^	1.34 ± 0.03^**##**^	1.07 ± 0.09^▲▲^
p62	1.00 ± 0.01	0.51 ± 0.02^**^	0.25 ± 0.02^**##**^	0.55 ± 0.02^△△^	0.77 ± 0.02^**##**^	0.95 ± 0.03^▲▲^

*Note*: **p < 0.01 versus normal group; ^##^ *p* < 0.01 versus insomnia group; ^△△^ *p* < 0.01 versus GAD67‐KD group; ^▲▲^ *p* < 0.01 versus GAD67‐OE group; the difference is statistically significant.

### 3.12 Protein expression patterns of GAD67 and pathway cytokines in rats

**FIGURE 7:**
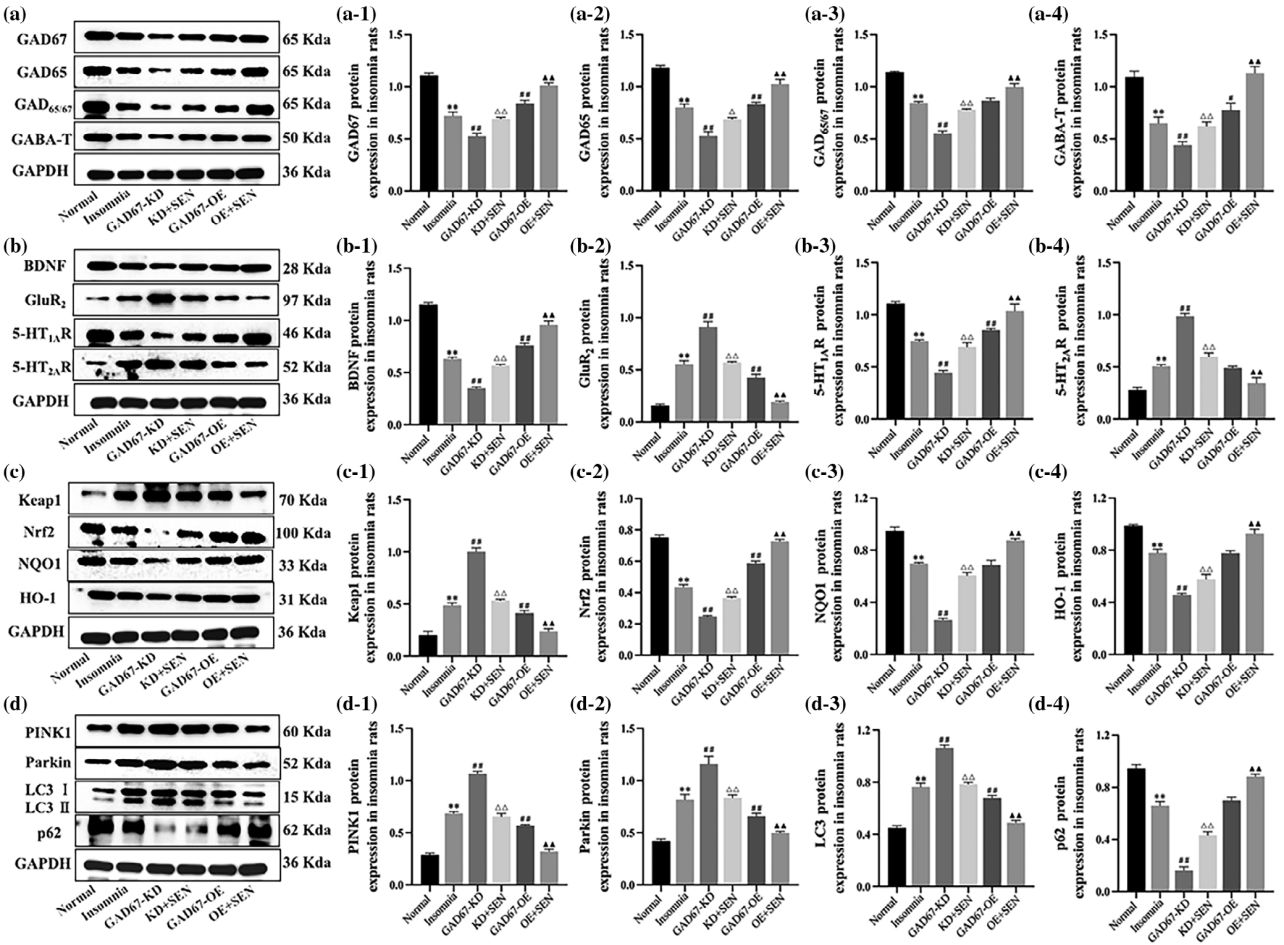
(a–d) Strip image of WB. Protein expression levels of (a‐1) GAD67, (a‐2) GAD65, (a‐3) GAD_65/67_, (a‐4) GABA‐T, (b‐1) BDNF, (b‐2) GluR_2_, (b‐3) 5‐HT_1A_R, (b‐4) 5‐HT_2A_R, (c‐1) Keap1, (c‐2) Nrf2, (c‐3) NQO1, (c‐4) HO‐1, and (d‐1) PINK1, (d‐2) Parkin, (d‐3) LC3, (d‐4) p62 in rats. GAPDH was used as an internal reference, and the experiments were repeated three times. Results are expressed as mean ± SD; **p < 0.01 versus normal group; ^#^ *p* < 0.05 or ^##^ *p* < 0.01 versus insomnia group; ^△^ *p* < 0.05 or ^△△^ *p* < 0.01 versus GAD67‐KD group; ^▲▲^ *p* < 0.01 versus GAD67‐OE group; the difference is statistically significant.

Western blotting was used to detect the protein expression of GAD67 and pathway cytokines in the rats after transfection with GAD67 lentiviral. As shown in Table 6, compared with the normal group, the protein expression levels of GAD67, GAD65, GAD_65/67_, GABA‐T, BDNF, 5‐HT_1A_R, Nrf2, NQO1, HO‐1, and p62 were significantly decreased in the insomnia group (*p* < 0.01), further decreased in the GAD67‐KD group (*p* < 0.01), and significantly increased after senegenin intervention (*p* < 0.01); the expression was significantly increased in the GAD67‐OE group (*p* < 0.01), and further increased after senegenin intervention (*p* < 0.01). In addition, protein levels of GluR_2_, 5‐HT_2A_R, Keap1, PINK1, Parkin, and LC3 were significantly increased in insomnia rats (*p* < 0.01), further increased in the GAD67‐KD group (*p* < 0.01), and decreased after senegenin intervention (*p* < 0.01); expression was significantly decreased in the GAD67‐OE group (*p* < 0.01) and further decreased after senegenin intervention (*p* < 0.01) (Figure 7).

**TABLE 6:** Protein expression patterns of GAD67 and pathway cytokines in rats.

Group	Normal	Insomnia	GAD67‐KD	KD + SEN	GAD67‐OE	OE + SEN
GAD67	1.11 ± 0.03	0.72 ± 0.04^**^	0.53 ± 0.03^**##**^	0.69 ± 0.02^△△^	0.84 ± 0.03^**##**^	1.01 ± 0.02^▲▲^
GAD65	1.18 ± 0.03	0.80 ± 0.03^**^	0.53 ± 0.04^**##**^	0.68 ± 0.02^△^	0.83 ± 0.02^**##**^	1.02 ± 0.05^▲▲^
GAD_65/67_	1.14 ± 0.01	0.84 ± 0.01^**^	0.55 ± 0.02^**##**^	0.78 ± 0.01^△△^	0.87 ± 0.02	1.00 ± 0.03^▲▲^
GABA‐T	1.09 ± 0.06	0.65 ± 0.06^**^	0.44 ± 0.03^##^	0.62 ± 0.04^△△^	0.78 ± 0.06^**#**^	1.13 ± 0.06^▲▲^
BDNF	1.15 ± 0.02	0.63 ± 0.02^**^	0.35 ± 0.01^**##**^	0.57 ± 0.01^△^	0.76 ± 0.02^**##**^	0.96 ± 0.03^▲▲^
GluR_2_	0.16 ± 0.01	0.55 ± 0.03^**^	0.91 ± 0.05^**##**^	0.57 ± 0.01^△△^	0.43 ± 0.03^**##**^	0.19 ± 0.01^▲▲^
5‐HT_1A_R	1.11 ± 0.02	0.75 ± 0.01^**^	0.44 ± 0.03^##^	0.69 ± 0.04^△△^	0.85 ± 0.01^**##**^	1.04 ± 0.07^▲▲^
5‐HT_2A_R	0.28 ± 0.02	0.51 ± 0.02^**^	0.99 ± 0.03^**##**^	0.60 ± 0.04^△△^	0.49 ± 0.02	0.35 ± 0.05^▲▲^
Keap1	0.20 ± 0.04	0.49 ± 0.02^**^	1.00 ± 0.04^**##**^	0.54 ± 0.02^△△^	0.41 ± 0.02^**##**^	0.24 ± 0.02^▲▲^
Nrf2	0.75 ± 0.02	0.44 ± 0.02*	0.25 ± 0.00^**##**^	0.36 ± 0.01^△△^	0.59 ± 0.02^**##**^	0.73 ± 0.02^▲^
NQO1	0.95 ± 0.03	0.70 ± 0.01^**^	0.27 ± 0.01^**##**^	0.61 ± 0.02^△△^	0.69 ± 0.03	0.88 ± 0.01^▲▲^
HO‐1	0.99 ± 0.01	0.78 ± 0.03^**^	0.46 ± 0.01^**##**^	0.58 ± 0.03^△^	0.78 ± 0.02	0.93 ± 0.03^▲▲^
PINK1	0.29 ± 0.02	0.69 ± 0.02^**^	1.07 ± 0.02^**##**^	0.66 ± 0.03^△△^	0.57 ± 0.01^**##**^	0.32 ± 0.02^▲▲^
Parkin	0.42 ± 0.02	0.82 ± 0.05^**^	1.16 ± 0.07^**##**^	0.83 ± 0.03^△△^	0.66 ± 0.03^**##**^	0.50 ± 0.01^▲▲^
LC3	0.45 ± 0.02	0.77 ± 0.03^**^	1.06 ± 0.02^**##**^	0.79 ± 0.01^△△^	0.68 ± 0.02^**##**^	0.49 ± 0.02^▲▲^
p62	0.94 ± 0.03	0.66 ± 0.03^**^	0.16 ± 0.03^**##**^	0.43 ± 0.03^△△^	0.70 ± 0.02	0.88 ± 0.02^▲▲^

*Note*: *p < 0.05 or **p < 0.01 versus normal group; ^#^ *p* < 0.05 or ^##^ *p* < 0.01 versus insomnia group; ^△^ *p* < 0.05 or ^△△^ *p* < 0.01 versus GAD67‐KD group; ^▲^ *p* < 0.05 or ^▲▲^ *p* < 0.01 versus GAD67‐OE group; the difference is statistically significant.

### 3.13 Cytokine protein expression in the brain tissue of rats

**FIGURE 8:**
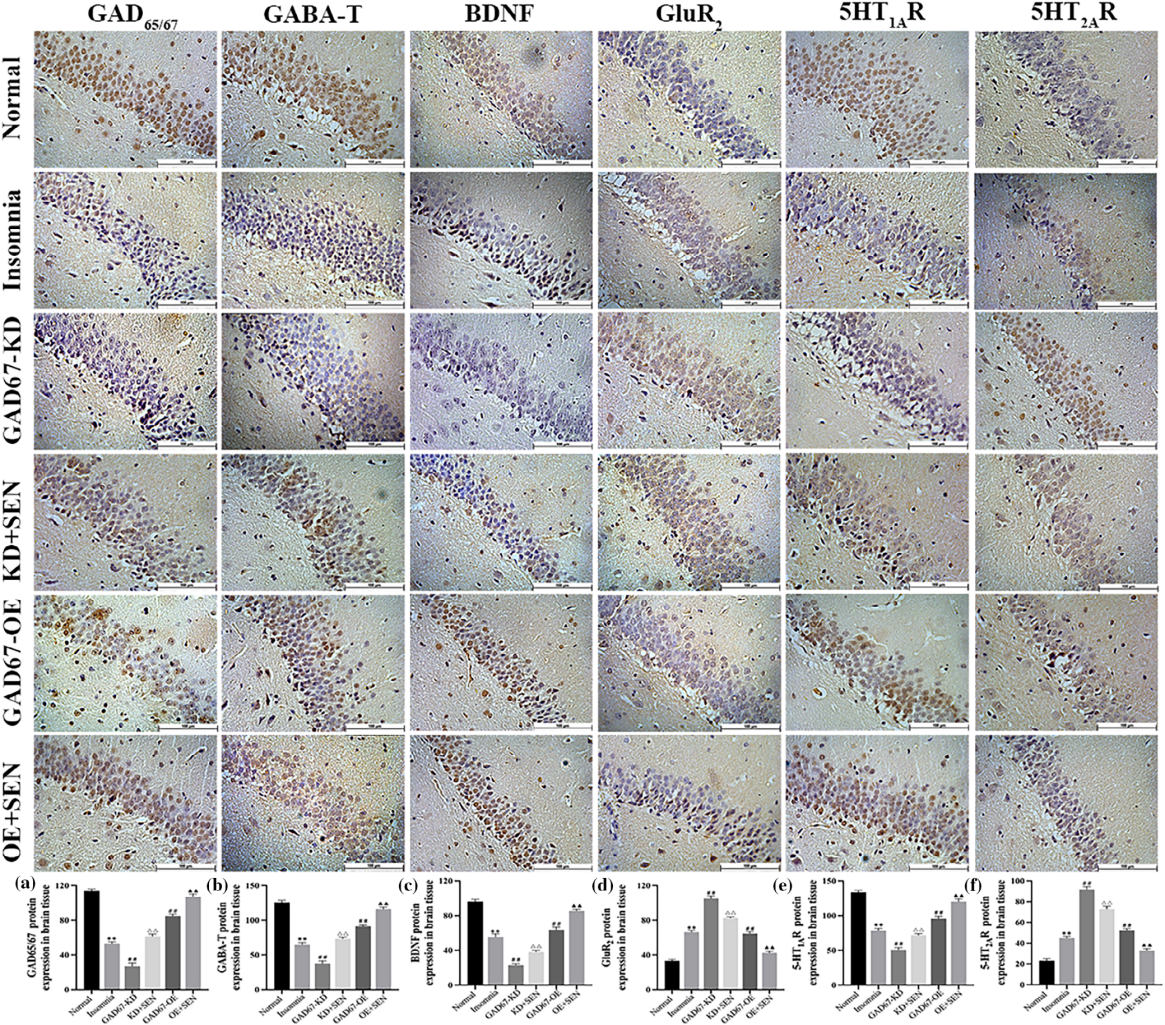
Protein expression levels of (a) GAD_65/67_, (b) GABA‐T, (c) BDNF, (d) GluR_2_, (e) 5‐HT_1A_R, and (f) 5‐HT_2A_R in the CA1 region of hippocampal tissue in rats was detected by IHC (200×). ** *p* < 0.01 versus normal group; ^##^ *p* < 0.01 versus insomnia group; ^△△^ *p* < 0.01 versus GAD67‐KD group; ^▲▲^ *p* < 0.01 versus GAD67‐OE group.

The protein expression of sleep factor in the CA1 region of rat hippocampus was detected by IHC. As shown in Figure 8, the expression of GAD_65/67_, GABA‐T, BDNF, and 5‐HT_1A_R protein levels was significantly decreased in CA1 region of hippocampus of insomnia rats (*p* < 0.01), further decreased after GAD67‐KD transfection (*p* < 0.01), and significantly increased after senegenin intervention (*p* < 0.01); the expression was significantly increased after GAD67‐OE transfection (*p* < 0.01) and further increased after senegenin intervention (*p* < 0.01). In addition, compared with the normal group, the protein levels of GluR_2_ and 5‐HT_2A_R in the CA1 region of hippocampus of insomnia rats were significantly increased (*p* < 0.01), further increased in the GAD67‐KD group (*p* < 0.01), and decreased after senegenin intervention (*p* < 0.01); the expression was significantly decreased in the GAD67‐OE group (*p* < 0.01) and further decreased after senegenin intervention (*p* < 0.01).

## 4 DISCUSSION

Insomnia is a common sleep disorder, and long‐term insomnia may lead to problems such as poor mental status, cognitive decline, and memory loss, which seriously affect the quality of life and health status of patients. In recent years, with deepening the understanding of TCM and the continuous development of scientific research, more and more studies have focussed on the role and effect of TCM e in treating insomnia. The results of RCTs of 1549 insomnia patients showed that TCM was significantly effective as an alternative therapy for insomnia (Li et al., 2020); the results of RCTs of 681 insomnia patients showed that XiaoYaoSan was effective in improving insomnia symptoms (Hu et al., 2021); and the results of RCTs of 1164 participants showed that Guizhi Gancao Longgu Muli Decoction could better treat insomnia compared with benzodiazepines, with fewer side effects (Chen, Chen, et al., 2022). In addition, Shuangxia decoction can enhance the expression of neurotransmitter 5‐HT_1_ in the hypothalamus and promote sleep (Sun et al., 2020); Ziziphi Spinosae regulates insomnia by activating GABA_A_R (Xiao et al., 2022); ginsenosides protect the central nervous system by inhibiting the activity of AChE, BChE, and BACE1 and the clearance of ONOO (−) to promote neuronal growth and anti‐ageing (Choi et al., 2016); BanXia‐YiYiRen may play a role in improving sleep by regulating the serotonergic pathway (Wang et al., 2022). In summary, TCM treatment of insomnia is effective and the side effects are small, seeking effective drugs to treat insomnia is an inevitable trend.

*Polygala tenuifolia*, as the first choice for the treatment of insomnia in TCM clinical practice, can shorten the time to fall asleep, prolong the time to sleep, and enhance the depth of sleep. It contains active components with multiple neuroprotective potentials associated with insomnia, such as anti‐inflammatory, antioxidant, and anti‐neuronal apoptosis, enhancing the central cholinergic system and promoting neuronal proliferation (Deng et al., 2020). Senegenin is the main active compound extracted from the rhizome of *Polygala tenuifolia* and is the central component of total saponins of *Polygala tenuifolia* and oleanolic acid pentacyclic triterpenoids, although *Polygala tenuifolia* enhances sleep by activating GABAergic systems or by the inhibition of noradrenergic systems and also mediates the balance of neurotransmitters by regulating serotoninergic systems to promote sleep (Chen et al., 2020), the mechanism by which its active components exert their efficacy has not been explored. Therefore, in this study, we investigated the mechanism related to insomnia in senegenin, the main active compound of *Polygala tenuifolia*, and explored the key pathways and targets of senegenin in the treatment of insomnia, providing a further theoretical basis for the treatment of insomnia with TCM.

GABA/Glu imbalance is an important mechanism leading to insomnia. GABA‐T, as a metabolic enzyme of GABA, metabolises GABA to pyruvate and acetamide to maintain the normal function of the nervous system; GAD67, as a key enzyme for the synthesis of GABA, activates GAD67 to catalyse the conversion and generation of Glu to GABA when neurons are stimulated, and inhibits neuronal excitability by binding to receptors. When the body is subjected to various factors leading to its reduced activity, GABA synthesis is insufficient and Glu accumulates too much, triggering an imbalance between inhibitory and excitatory neurotransmitters, resulting in insomnia (Bruni et al., 2021). It has been shown that GABA/Glu imbalance in mice leads to impaired excitation/inhibition balance of neurons in the central nervous system of the brain, which triggers neurological disorders (El‐Khoury et al., 2014); the expression level of GABA in brain tissue is positively correlated with changes in sleep–wake depth (Weber et al., 2015); the GABA‐T content is significantly reduced in insomnia mice (Jeon et al., 2015), and increasing GABA‐T content in brain and serum can prolong RNEM and REM and promote sleep (SiSi et al., 2022). In addition, GAD67 interacts with GABA to maintain normal sleep rhythms (Cho et al., 2010); up‐regulation of GAD_65/67_ protein expression is able to activate the GABAergic system and increase NREM sleep (Shah et al., 2014). The results of this study showed that GAD67 expression was decreased in insomnia patients, GluR_2_ content was increased and GABA‐T content was decreased in insomnia rats, and senegenin intervention could increase GAD67 and GABA‐T content. In addition, GAD_65/67_ decreased in insomnia rats after GAD67 knockdown, which could activate GABA‐T by up‐regulating GAD_65/67_ by overexpressing GAD67, consistent with previous findings.

Newer studies have shown that changes in GABAergic neuronal activity in the hippocampus can mediate sleep changes (Katsuki et al., 2022), and the GABAergic system regulates sleep by releasing GABA to inhibit neuronal activity, and GAD67 is a key enzyme in the synthesis of GABA. Therefore, we made the hypothesis: can GAD67 and the hippocampus act directly? Will senegenin continue to mediate a signalling pathway through GAD67 to regulate insomnia after intervention? To test this hypothesis, we first performed an EGG/EMG analysis of the insomnia rat model, and the results showed that the wake period in the insomnia group was significantly increased compared with the normal group of rats in the light–dark stage, and the NREM and REM periods were significantly reduced, which indicated that we had successfully established the insomnia model. We performed behavioural tests in rats and showed that insomnia rats developed dysfunction in learning memory and spatial exploration, which could be ameliorated by the intervention of senegenin. Through HE staining, we found that the pathological changes in the CA1 region of the hippocampus were particularly obvious, and GAD67 was significantly decreased in the insomnia group, overexpression of GAD67 would increase its expression, and senegenin was further up‐regulated after intervention, which indicated that senegenin could improve insomnia by regulating GAD67, and was closely related to hippocampal tissue. The expression of sleep factors in the CA1 region of the hippocampus is even more indicative of this condition.

The Keap1/Nrf2 signalling pathway is a key pathway against stress oxidation in the body and can protect neural cells against oxidative stress injury. Studies have found that oxidative stress parameters are significantly up‐regulated in insomnia patients compared with controls (Gulec et al., 2012); increasing antioxidant capacity in the brain can reduce oxidative stress caused by insomnia (Zheng et al., 2023). In addition, the altered signal intensity of the Keap1/Nrf2 pathway similarly regulates altered expression levels of downstream factors NQO1 and HO‐1. Chen's results showed that senegenin exerts neuroprotective effects by regulating Nrf2/HO‐1 pathways, reducing oxidative stress, and inhibiting inflammation and apoptosis (Chen, Yang, et al., 2022), which tends to be consistent with the results that senegenin can improve insomnia by regulating Keap1/Nrf2/NQO1/HO‐1 in this experiment. In this study, we showed that Keap1 expression was increased and Nrf2, NQO1, and HO‐1 expression was decreased in insomnia rats, and after senegenin intervention, Keap1 expression was decreased and Nrf2, NQO1, and HO‐1 expression was increased. In addition, we transfected GAD67 into PC12 cells and rats, Keap1 expression was increased and Nrf2, NQO1, and HO‐1 expression was decreased in the GAD67‐KD group, Keap1 expression was decreased and Nrf2, NQO1, and HO‐1 expression was increased in the GAD67‐OE group, and cytokine expression could be regulated in a significant correlation after senegenin intervention. The above results indicate that senegenin can regulate the Keap1/Nrf2 signalling pathway to improve insomnia by mediating GAD67.

The PINK1/Parkin signalling pathway is a key pathway in the body to regulate mitophagy and can regulate mitochondrial function‐specific autophagy (Duan et al., 2022). Mitochondria play an important role in neuroprotection and anti‐neurodegeneration (Kerr et al., 2017), and when damaged mitochondria cannot be removed in time, they lead to neuronal apoptosis, which leads to the occurrence of neurological diseases such as insomnia (Lou et al., 2020); mitophagy is also an important factor in the pathogenesis of insomnia and can be used as a biomarker for related neurological diseases (Palagini et al., 2022), and oxidative stress is involved in regulating the expression of mitophagy (Ding et al., 2019; Kong et al., 2021; Li et al., 2023). It has been shown that the PINK1/Parkin pathway has a central role in memory improvement in the brain (Holland et al., 2020), and its signalling dysregulation can cause mitochondrial damage in the brain and promote oxidative stress response, which causes neuronal apoptosis (Aman et al., 2021; Cai & Jeong, 2020). The results of this study showed that PINK1, Parkin, and LC3 expression was increased and p62 expression was decreased in insomnia rats, and senegenin intervention decreased the expression of PINK1, Parkin, and LC3 and increased the expression of p62. In addition, knockdown of GAD67 upregulated PINK1, Parkin, and LC3 and downregulated p62 expression, and overexpression of GAD67 downregulated PINK1, Parkin, and LC3 and upregulated p62 expression in GAD67‐transfected rats, which could significantly regulate cytokine expression after senegenin intervention. Although the above results suggest that senegenin can regulate insomnia by mediating GAD67 to regulate the PINK1/Parkin signalling pathway, interestingly, the mRNA and protein expression of PINK1, Parkin, and LC3 were inconsistent in GAD67‐transfected PC12 cells, and the mRNA expression of PINK1, Parkin, and LC3 was increased in the GAD‐OE group, but the protein expression was decreased, which we hypothesise is due to changes in the translational levels of certain organelles and requires further exploration.

In summary, the pathogenesis of insomnia is complex, and although CBT‐I and drug therapy have advantages, they cannot be used popularly in the treatment of a wide range of insomnia morbidity due to problems such as medical human resources and drug dependence. In recent years, it has become a hot topic to explore the mechanism of insomnia at the genetic level. Therefore, in this study, GAD67 lentivirus was transfected at the epigenetic level in vitro and in vivo to explore the mechanism of senegenin in regulating insomnia through the GAD67‐mediated Keap1/Nrf2/Parkin/PINK1 pathway. Although this study provides part of the rationale for senegenin in the treatment of insomnia in molecular mechanisms, other mechanisms of action by which it exerts its efficacy have not been investigated and require further exploration.

## 5 CONCLUSION

GAD67 is negatively correlated with sleep–wake rhythm and can regulate Keap1/Nrf2/Parkin/PINK1 expression, and senegenin can further regulate Keap1/Nrf2/Parkin/PINK1 by mediating GAD67, thus playing a regulatory role in the development of insomnia.

## AUTHOR CONTRIBUTIONS

**Honglin Jia:** Data curation; methodology; validation; formal analysis; funding acquisition; writing – original draft. **Xu Chen:** Data curation; methodology; software; validation; formal analysis; writing – original draft. **Zhengting Liang:** Methodology; project administration; software; funding acquisition. **Ruining Liang:** Methodology; project administration; software; supervision. **Jinhong Wu:** Formal analysis; software; investigation; supervision. **Yanling Hu:** Resources; investigation; software. **Wenjun Cui:** Investigation; software; supervision. **Xingping Zhang:** Supervision; conceptualization; funding acquisition; project administration; writing – review and editing.

## FUNDING INFORMATION

This work was supported by the National Natural Science Foundation of China (No. 82160873; 81960837), Sponsored by Natural Science Foundation of Xinjiang Uygur Autonomous Region (No. 2022D01D48; No. XJ2024G163).

## CONFLICT OF INTEREST STATEMENT

The authors declare that they have no conflicts of interest.

## CONSENT FOR PUBLICATION

All the authors have read the contents of the manuscript and have provided their consent to publish the data.

## Supporting information


**DATA S1.** Supporting information.

## Data Availability

The data that support the findings of this study are available from the corresponding author upon reasonable request.
