# Supplementary material for: Senegenin regulates the mechanism of insomnia through the Keap1/Nrf2/PINK1/Parkin pathway mediated by GAD67
Source: J Sleep Res. 2024 Oct 8;34(3):e14354. doi: 10.1111/jsr.14354 (PMC12069745; doi:10.1111/jsr.14354)
Supplement: Supplementary file 1 — DATA S1. Supporting information. [file JSR-34-e14354-s001.docx]

The sequence of lentiviral overexpression of rat GAD67 was as follows:

ATGGCCTTCTTGTTGACTACCCGACGGCTGGTCTGCAGTTCCCAGAAAAACCTCCACCTCTTCACACCTGGATCCAGATACATCAGCCAAGCTGCTGCCAAAGTTGACTTTGAGTTTGATTATGATGGACCACTCATGAAGACAGAAGTCCCGGGCCCTAGATCTCAGGAGCTAATGAAACAGCTGAACACAATCCAGAATGCAGAGGCCGTGCACTTTTTCTGCAACTACGAAGAGAGCCGAGGCAACTACCTCGTGGACGTGGATGGCAACCGCATGTTGGACCTGTATTCTCAGATCTCCTCTGTACCCATCGGTTACAACCATCCGGCTCTGGCGAAACTCGTTCAACAGCCTCAAAACGCGAGCACTTTCATCAACAGACCTGCCCTGGGCATCCTGCCTCCAGAGAACTTTGTGGACAAGCTCCGGGAGTCCTTGATGTCGGTGGCGCCCAAAGGCATGTGTCAGCTCATCACGATGGCCTGCGGGTCCTGCTCCAATGAGAATGCATTCAAGACCATCTTCATGTGGTACCGGAGTAAAGAACGAGGTCAGAGAGGTTTCTCCAAAGAGGAGCTGGAGACTTGCATGGTTAACCAGAGTCCTGGATGCCCAGACTACAGCATCCTCTCCTTCATGGGTGCTTTCCACGGGAGGACCATGGGTTGCTTAGCGACCACACACTCCAAAGCAATTCACAAGATTGACATCCCTTCCTTTGACTGGCCCATTGCTCCATTCCCACGGCTGAAATATCCCCTGGAGGAGTTTGTGACGGACAATCAGCAAGAGGAGGCCCGCTGTCTAGAAGAGGTGGAGGATCTAATTGTGAAATATCGGAAAAAGAAGAGAACAGTGGCTGGGATCATCGTGGAGCCCATCCAGTCCGAAGGTGGAGACAACCACGCATCAGATGACTTCTTCCGGAAGCTGAGAGACATAGCCAGGAAGCATGGCTGTGCCTTCTTGGTGGACGAGGTTCAGACTGGAGGAGGCTGTACAGGCAAGTTCTGGGCCCATGAACACTGGGGCTTGGATGACCCAGCCGACGTGATGTCGTTCAGCAAGAAGATGATGACTGGGGGCTTCTTCCACAAGGAGGAGTTTCGACCAAGTGCTCCTTACCGGATCTTCAACACCTGGCTGGGGGACCCATCCAAGAACTTGCTGCTGGCTGAGGTCATCAACATCATCAAGCGGGAAGACCTGCTCAACAACGTGGCCCATGCCGGGAAGACCCTACTGACCGGGCTGCTGGACCTCCAGGCCCAGTACCCCCAGTTCGTCAGCCGGGTGAGGGGACGAGGCACCTTCTGTTCCTTCGACACTCCCGACAAAGCCATACGGAATAAACTCATCCTAATTGCCAGGAACAAAGGTGTGGTACTGGGGGGCTGCGGTGACAAATCCATACGTTTCCGTCCCACGCTGGTCTTCAGGGATCACCATGCCCACTTGTTCCTCAACATTTTCAGTGGTATCTTAGCAGACTTCAAGTAA
